# Study on Bamboo Longitudinal Flattening Technology

**DOI:** 10.3390/polym14040816

**Published:** 2022-02-20

**Authors:** Tiancheng Yuan, Tao Zhang, Yaqian Huang, Yifei Wu, Xinzhou Wang, Yanjun Li

**Affiliations:** 1 Jiangsu Co-Innovation Center of Efficient Processing and Utilization of Forest Resources, Nanjing Forestry University, Nanjing 210037, China; ytc_njfu@163.com (T.Y.); zt@njfu.edu.cn (T.Z.); huangyaqian1997@163.com (Y.H.); wuyifei_njfu@163.com (Y.W.); xzwang@njfu.edu.cn (X.W.); 2 Bamboo Engineering and Technology Research Center, State Forestry and Grassland Administration, Nanjing 210037, China

**Keywords:** bamboo flattening technology, parenchyma cell, chemical composition, hardness

## Abstract

In this paper, we introduced a bamboo longitudinal flattening technology and analyzed the effects of the softening–flattening process on the physical and mechanical properties of moso bamboo. This is a newer bamboo processing technology that can enhance the utilization and reduce pollution compared with traditional bamboo-based products. Results showed that the parenchyma cells distorted and compacted due to the flattening process. The hemicellulose and cellulose content decreased, while the content of lignin presented an increasing tendency. As expected, the dimensional stability of moso bamboo enhanced due to the decrement of hemicellulose. The softening–flattening process positively contributed to the micro-mechanical properties of treated bamboo specimens. For example, the hardness and modulus of elasticity of the untreated bamboo sample increased from 0.58 and 15.7 GPa to 0.8 and 17.5 GPa, respectively. In addition, the changes in cellulose crystallinity and mechanical properties were also investigated in this paper. The cellulose crystallinity increased from 37.5% to 43.2%, significantly. However, the modulus of rupture of the flattened bamboo board decreased from 9000 to 7500 MPa due to the grooves made by the flattening roller. The MOE of flattening bamboo board showed the same decreasing tendency.

## 1. Introduction

As an abundant biomass material, bamboo has attracted more attention due to its short growth cycle, excellent mechanical property, and easy harvest [1,2,3,4]. In addition, bamboo can be widely applied in the areas of decoration, furniture, construction, and so on. However, the inner drawbacks restrict bamboo’s application, such as dimensional instability, hydrophilic property, and ease of decay [4,5,6,7,8]. For practical application products, bamboo scrimber, laminated bamboo board, bamboo rotary cutting veneer, oriented strand board, fiberboard, and ply bamboo have received increasing attention in the past decades as a substitute for wood materials. For bamboo culm, nodes and internodes are the basic constituents. Thus, bamboo cannot be processed like wood because of its different structure. Furthermore, it is hard to obtain a large original bamboo board with elegant texture through the saw cutting process. Bamboo flattening technology is a useful technology for solving this difficulty [9].

Bamboo culms consist of hemicellulose, cellulose, lignin, ash, and so on [10]. The main chemical compositions mentioned above are containing amorphous phases. When exposed to high temperature and high-pressure conditions, these chemical components exhibit viscoelastic and plastic behaviors. Normally, scientists use glass transitions to describe this thermal softening behavior. The polymers become rubbery and soften when the temperature is above glass transitions [11,12,13,14]. Bamboo culms can be flattened into flattened bamboo board in this “rubbery” state. Like wood materials, bamboo can be softened by heat treatment medium (hot air, hot water, high-frequency heating, microwaves, and hot oil) and chemical agents (NaHCO_3_, NaOH, and KOH). However, treatment with chemical agents is not environmentally friendly and affects the natural and sustainable character of bamboo. Through reviewing some reported studies and patent [11,12,13,14,15,16,17,18] and after visiting some industries in China, bamboo softened by saturated steam in a sealed equipment is shown to be most the eco-friendly and cost-efficient method now. Compared with traditional softening treatment mediua (oil, hot water, hot air, etc.), saturated steam can provide a mild environment consisting of high moisture content and high pressure. 

**Figure 1:**
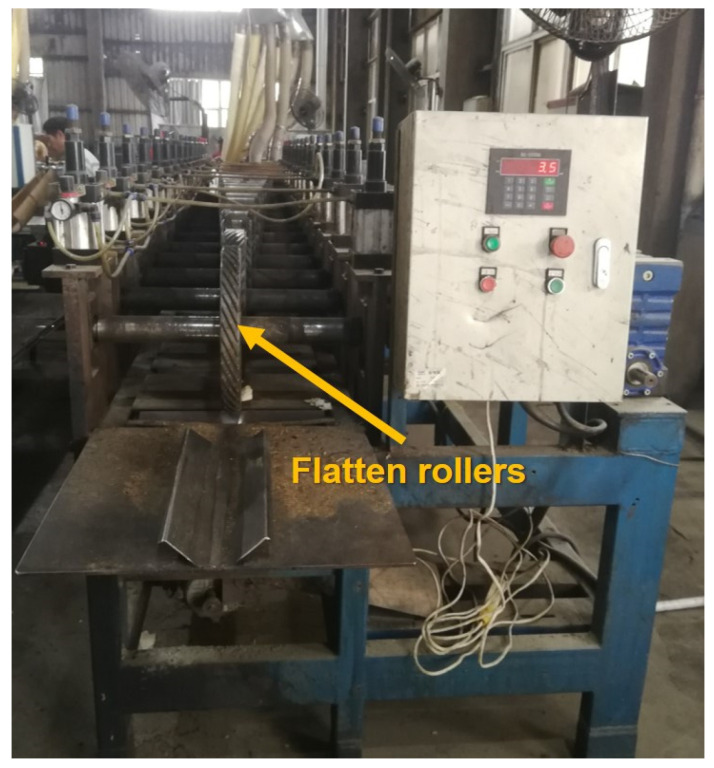
The bamboo longitudinal flattening machine.

Bamboo flattening technology has been developed over 30 years. In 1988, Zhang first reported bamboo flattening technology. Zhang softened bamboo culms through hot water and then flattened half-tubular bamboo culms via a hot press. Although the flattened bamboo board was obtained, unfortunately, many deep cracks were shown in the bamboo surface. Parkkeeree and his colleagues reported a flattening process on black sweet bamboo (*Dendrocalamus asper*) with hot oil [15]. Bamboo culms were flattened by two plates. However, the efficiency of the process was very low. Liu et al. did flattening tests on 6-year-old moso bamboo (*phyllostachys pubescens*) with a special device [16]. The bamboo splices were flattened by applying the horizontal forces. However, the process is controlled manually with very low efficiency, and the width of the flattened bamboo board is limited. In recent years, some scholars reported “notched flattening technology” [17]. “Notched flattening technology” can flatten bamboo culms through making non-penetrating indentations in order to increase the circumference of the inner layer so that the softened bamboo culms can be flattened without cracks. However, this “notched flattening technology” cannot flatten long bamboo culms because of the different thickness of the bamboo wall along the longitudinal direction. Many entrepreneurs and scientists are working on the invention of bamboo longitudinal flattening technology, which can broaden the field of application of flattened bamboo board. Recently, a device that can flatten long bamboo culms has been created by scientists (Figure 1). This bamboo flattening equipment consists of a set of rollers with knives. The long bamboo culms can be flattened gradually with the increasing width of flattening rollers. However, few literatures focused on this newer bamboo longitudinal flattening technology.

In this paper, we reported a bamboo longitudinal flattening technology and focused on the effect of the softening–flattening process on the micro-morphology, chemical composition, physical properties, and mechanical properties of bamboo via X-ray diffraction (XRD), scanning electron microscopy (SEM), Fourier transform infrared (FTIR), the wet chemistry method, and nanoindentation (NI). In addition, we proposed the best softening parameters by means of flatten tests at different temperatures and times.

## 2. Materials and Methods

### 2.1. Materials

Six-year-old moso bamboo (*Phyllostachys heterocycla*) was collected from Lishui city, Zhejiang, China. The initial moisture content of moso bamboo was 100%, and there were no obvious defects in the bamboo surface.

### 2.2. Manufacturing Process of Crack-Free Flattened Bamboo Board

**Figure 2:**
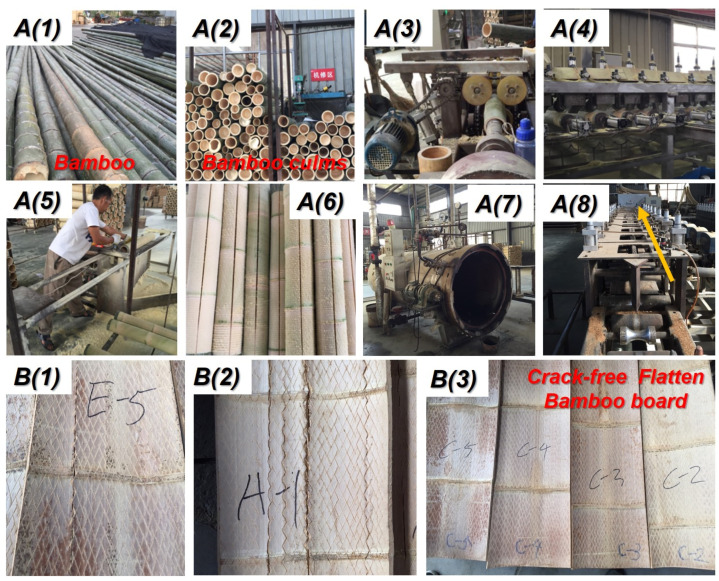
Manufacturing process of crack-free, flattened bamboo board: **A(1)** bamboo; **A(2)** bamboo culms; **A(3)** remove yellow bamboo and inner nodes machine; **A(4)** remove green bamboo machine; **A(5)** splitting bamboo culms; **A(6)** the bamboo culms after splitting process; **A(7)** saturated steam heat treatment device; **A(8)** bamboo flattening machine; **B(1,2)** flatten bamboo board with obvious cracks; **B(3)** obtained successful crack-free flatten bamboo board through longitudinal flattening technology.

Firstly, we sawed the natural bamboo into bamboo culms with the same length. Then, the bamboo tubes passed through the special equipment to remove the green bamboo and yellow bamboo (Figure 2A(2–4)). The combined milling cutter was inserted into the bamboo tube, and the punch then broke through the partition. Next. the bamboo tube was supported and rotated by four friction wheels with an opposite direction to the milling cutter. Then. the green bamboo and yellow bamboo were removed. After removing the green bamboo and yellow bamboo, the worker made a sawing slit in the longitudinal direction of the bamboo culms so that the saturated steam can easily pass through the bamboo tube (Figure 2A(5)). As shown in Figure 2A(6,7), the slitted bamboo tubes were waiting for saturated steam heat treatment at different temperatures and times in sealed equipment (Figure 2A(8)). After the flattening process, the cracked flattened bamboo board (Figure 2B(1,2)) and crack-free bamboo can be obtained (Figure 2B(3)). The successful ratio of flattened bamboo board during the softening–flattenomg process is shown in Table 1. According to Table 1 (successful ratio of flattened bamboo board), we considered 180 °C and 6–8 min as the well-softened parameters for manufacturing crack-free, flattened bamboo board.

### 2.3. Observation of Microstructure of Bamboo

The cross-sections of natural bamboo and the softened bamboo were sliced by a knife. Then, samples were placed in a vacuum environment followed by spray-gold treatment until the surface of samples had conductivity. The microstructure of bamboo and the softened bamboo were observed by scanning electron microscopy (SEM) (Quanta 2000, Tokyo, Japan) at an accelerating voltage 15 kV.

### 2.4. Wet Chemistry Method

The main chemical composition (cellulose, hemicellulose, and lignin) content of the samples were tested according to NREL’S LAPS [18,19,20,21]. For details, the concentration of monosaccharides in the supernatants were determined using a high performance liquid chromatography (HPLC) system (LC-2800, Agilent Technologies Inc., Palo Alto, CA, USA) with a refractive index detector, and H_2_SO_4_ solution was used as the eluent at a flow rate of 0.6 mL/min.

### 2.5. Measurement of Cellulose Crystallinity Degree

The cellulose crystallinity of untreated bamboo, softened treated bamboo, and flattened treated bamboo were measured through the X-ray diffraction (XRD) spectra combined with JADE software. Untreated bamboo, softened treated bamboo, and flattened treated bamboo were ground into powder (80−100 mesh) and dried under a vacuum at 80 °C for 12 h to make tests. The XRD spectra were recorded by a combined multifunctional horizontal X-ray diffractometer (Ultima IV, Tokyo, Japan) with a rate of 2 °C/min ranging from 5 to 40 °C. The crystallinity degree can be calculated as below [22,23]:

CrI = (I_002_ − I_am_)/I_002_ × 100% (1)

where CrI represents the crystallinity index, I_am_ represents the minimum intensity of the amorphous, and I_002_ represents the maximum intensity of the diffraction.

### 2.6. FTIR

The natural bamboo and softened treated bamboo were powdered into 100−200 mesh size and dried under vacuum at 80 °C for 12 h. The powder from different samples was used to making Fourier-transform infrared (FTIR) spectroscopy analysis by Nicolet iS50 FT-IR spectrometer (Thermo Fisher Scientific, Waltham, MA, USA). Data in the wave number range of 4000–500 cm^−1^ were collected in ATR mode with 64 scans and a resolution of 4 cm^−1^.

### 2.7. EMC, Shrinkage, MOE, and MOR of Bamboo Samples after Softening–Flattening Process

Moso bamboo culms and flatten bamboo boards (140, 150, 160, 170, and 180 °C and 4, 6, and 8 min) were used as raw materials. The equilibrium moisture, air dry shrinkage ratio (radial and tangential direction), modulus of elasticity, and modulus of rupture were tested according to GB/T 15780-1995 “Experimental method for physical and mechanical properties of bamboo”. The bamboo samples with the dimensions of 160 × 10 × 8 mm^3^ (Longitudinal × tangential × radial) were prepared for mechanical testing. Twelve repeated samples of each experimental set were tested in the three-point bending test to analyze the modulus of rupture (MOR) and modulus of elasticity (MOE).

### 2.8. Nanoindentation Method

**Figure 3:**
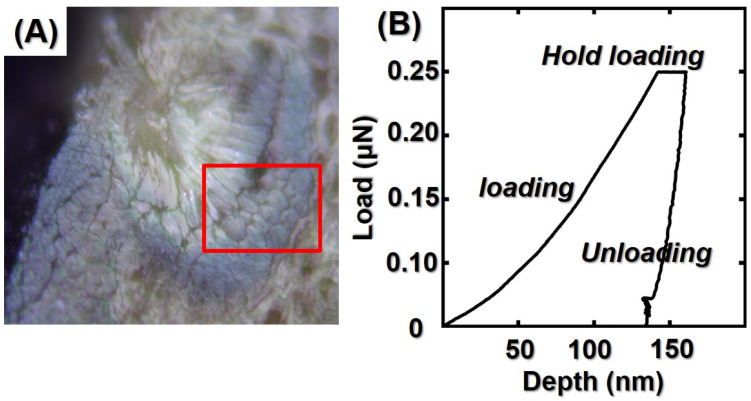
(**A**) Test location of bamboo cell walls; (**B**) typical nanoindentation (NI) load–depth curves.

Bamboo samples were cut into smaller blocks of 5 × 5 × 10 mm^3^, and the transverse section of the blocks was polished by a diamond knife (Micro Star Inc., Huntsville, TX, USA) in order to obtain areas of fibers with a surface roughness of less than 10 nm. The cell areas were imaged with an AFM built into a nanoindenter (Ti800, Hysitron Inc., Minneapolis, MN, USA), as shown in Figure 3. The test location was chosen at the secondary wall layer of the cell wall, and six cells were randomly selected on each specimen for testing. The test was performed in a three-stage constant rate loading and unloading mode (loading/holding/unloading for 5 s) with a maximum load of 400 μN. The test location was rescanned at the end of the test to obtain indentation images, and 30 valid indentation points were selected. The loading–displacement curves of these valid points were statistically analyzed according to the Oliver and Pharr method [24,25,26]. The modulus of elasticity of bamboo cell walls can be calculated as below:

(2) $$H=\frac{P_{max}}{A}\;$$

where *P_max_* is the peak load, and *A* is that the projected contact space of the indents at peak load. The hardness of different treated bamboo specimens can be calculated as follows:

(3) $$Er=\frac{\sqrt{\pi }}{2\beta }\frac{S}{\sqrt{A}}$$

where *Er* is the combined elastic modulus of both the sample and indenter, *S* is initial unloading stiffness, and *β* is a correction factor correlated to indenter geometry (*β* = 1.034).

### 2.9. Statistical Analysis

Tukey’s tests were used to distinguish the differences between the untreated bamboo samples and treated groups via Statistical software SPSS [23]. In addition, 12 replicates were used to measure means for modulus of elasticity and hardness. There were three replicates used for cellulose crystallinity degree, main chemical component, and chemical groups in bamboo samples. The images in this article were drawn by origin v9.0 [24]. Different capital letters represent the significant difference between heat treatment groups (*p* < 0.05).

## 3. Results and Discussion

### 3.1. SEM Analysis

**Figure 4:**
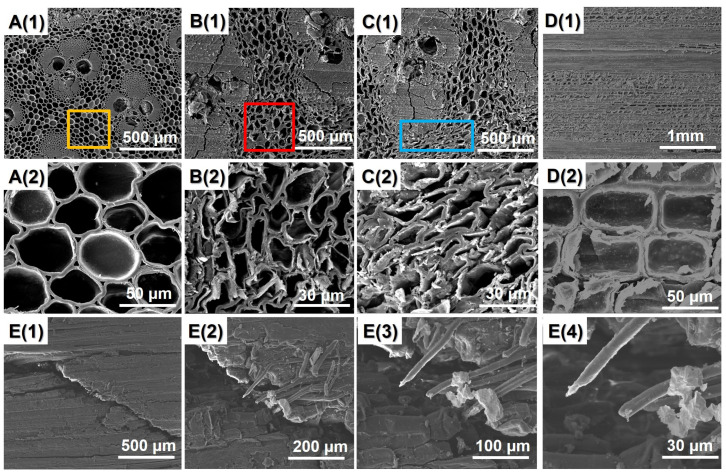
SEM images of different bamboo samples: **A(1,2)** untreated bamboo sample; **B(1,2)** softened treated bamboo samples (180 °C/8 min); **C(1,2)** flattened bamboo board at 180 °C/8 min; **D(1,2)**: tangential section images of untreated bamboo samples; **E(1–4)** tangential section images of flattened bamboo board.

The SEM images of bamboo samples before and after the softening–flattening process are shown in Figure 4. Figure 4A presents the cross-section of the untreated bamboo specimen. As shown in Figure 4A, the parenchyma cell wall and vascular bundles of untreated bamboo is round and smooth. SEM observation reveals that the bamboo lumen volumes are gradually decreased during the saturated steam heat treatment. In other word, the bamboo parenchyma cell walls deformed and distorted due to the external pressure and high temperature. This is because the high pressure and high-temperature condition degraded the main chemical composition in the bamboo cell walls, resulting in this deformation [27,28,29]. The flattening process compressed the parenchyma cell without obvious cell wall failure in comparison to the untreated bamboo specimen due to the viscoelastic nature of bamboo. During the flattening process, the bamboo culms exhibit rubbery behavior so that the bamboo culms can be easily flattened without marked damage. However, compared with tangential section of the untreated bamboo sample (Figure 4D(2)), the bamboo longitudinal flattening technology made obvious cuts on the inner surface of bamboo culms (Figure 4E(2)).

### 3.2. Effect of Saturated Steam Heat Treatment on the Relative Cellulose Crystallinity, Chemical Composition, and Chemical Functional Groups of Bamboo Samples

**Figure 5:**
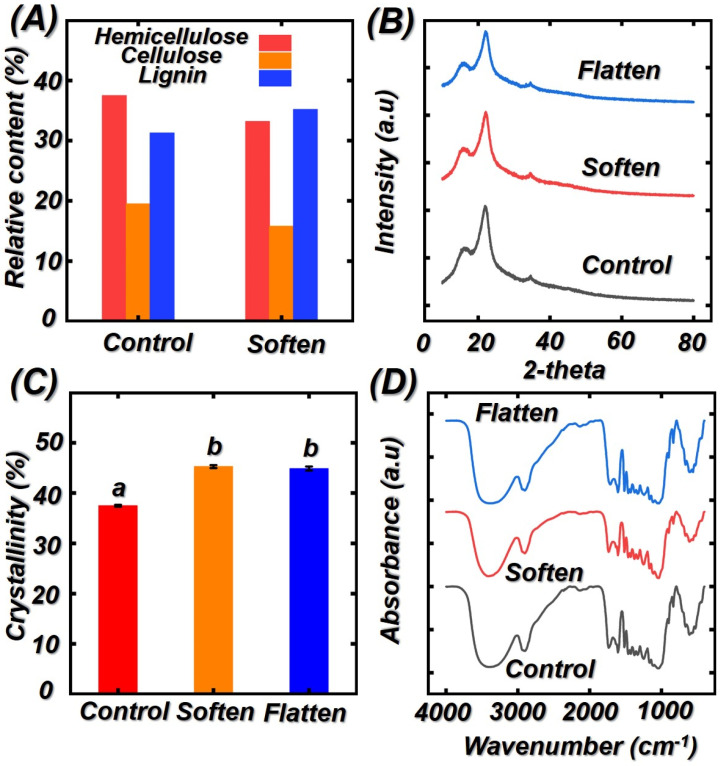
The change in chemical composition, cellulose crystallinity, and chemical groups of different bamboo samples: (**A**) hemicellulose, cellulose, and lignin; (**B**) XRD curves; (**C**) relative crystallinity degree; (**D**) FTIR curves of different bamboo samples. Different capital letters represent the significant difference between heat treatment groups (*p* < 0.05). The error bar in the picture represents the standard deviation.

As shown in Figure 5A, the initial hemicellulose, cellulose, and lignin of the control are 19.5%, 37.5%, and 31.3%, respectively. It can be seen from Figure 5A that the softening process has a significant effect on the change of hemicellulose, cellulose, and lignin content. For example, the hemicellulose and cellulose decreased from 19.5% and 37.5% to 15.8% and 33.2%. As a short chain, hemicellulose contains many amorphous polysaccharides with acetyl groups. During the saturated steam heat treatment process, more acetic acid was released from acetyl groups, resulting in the degradation of amorphous polysaccharides in hemicellulose. Thus, the hemicellulose content presented a decreasing tendency. Different from decreasing hemicellulose and cellulose content, the lignin content increased from 31.3% to 35.2%. The lignin condensation and cross-link reactions may contribute to this change. In addition, the increased lignin content played a positive role in the stiffness and strength of bamboo cell walls [23,30,31,32].

To further investigate the cellulose crystallinity degree of treated bamboo samples after saturated steam heat treatment, XRD curves were analyzed, and the corresponded XRD curves and calculated relative cellulose crystallinity degree were presented in Figure 5B,C. The crystallinity index (CrI) increased from 37.5% to 43.2% after saturated steam heat treatment. The increment of CrI can be attributed to the decomposition of hemicellulose. In addition, the rearrangement of the amorphous regions in cellulose may also lead to the increment of CrI. According to previous literature [33,34,35], the strength in the amorphous region is lower than that of crystalline region in cellulose. Thus, the decomposition of the amorphous region can enhance the mechanical properties of bamboo cell wall. Although the soften process increased the CrI significantly; however, results from statistical analysis showed that there is no significant enhancement in CrI after the flattening process because the flattening process is a physical change.

The influence of saturated steam heat treatment on the chemical groups of bamboo samples were analyzed by FTIR. Figure 5D presented the FTIR curves of untreated and soften treated bamboo samples. The main chemical structure of bamboo unchanged too much, while the intensities of some peaks were decreased. The intensity of peaks at 1730, 1159 (C−O−C stretching vibration), and 1318 cm^−1^ (O−H stretching vibration) decreased, indicating the decomposition of hemicellulose. Under the hydrothermal environment, the acetyl group hydrolyzed to form acetic acid. Thus, the number of carbonyl C=O bands (1630 cm^−1^) decreased due to the formation of acetic acid so that the cellulose content decreased. The peak at 1590 cm^−1^ represents the C=C stretching vibrations in lignin. As compared to the control groups, the intensity of 1590 cm^−1^ increased, which confirmed the condensation reaction of lignin during saturated steam heat treatment [36,37,38,39].

### 3.3. Equilibrium Moisture Content (EMC) and Shrinkage of Bamboo after Saturated Steam Heat Treatment and Flattening Process

**Figure 6:**
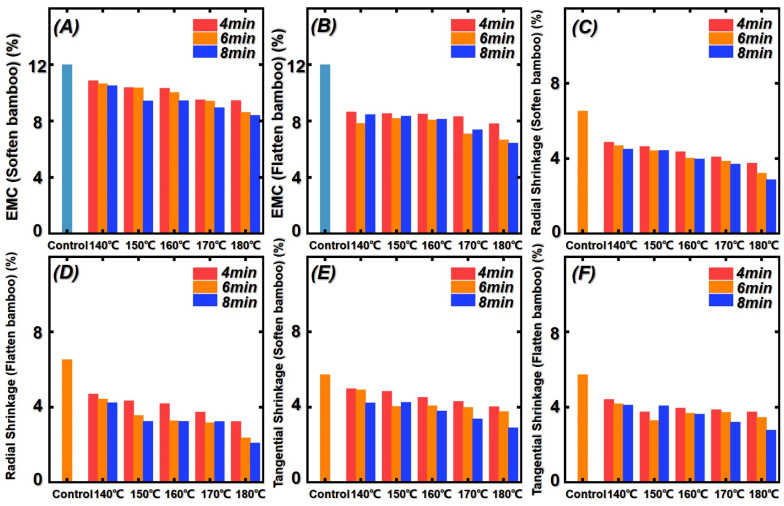
Physical properties of untreated, softened treated, and flattened bamboo samples: (**A**) EMC (softened bamboo); (**B**) EMC (flattened bamboo); (**C**) radial shrinkage ratio (softened bamboo); (**D**) radial shrinkage ratio (flattened bamboo); (**E**) tangential shrinkage ratio (softened bamboo); (**F**) tangential shrinkage ratio (flattened bamboo).

To explore the effect of the softening–flattening process, the physical properties of bamboo samples, equilibrium moisture content (EMC) and the change of radial and tangential shrinkage, were tested. The results of EMC and radial and tangential shrinkage are presented in Figure 6. As shown in Figure 6, the initial EMC and radial/tangential shrinkage were 11.99%, 6.53%, and 5.73%, respectively. The EMC of treated bamboo samples decreased with the increasing treatment temperature and time. In other words, the hygroscopicity decreased due to the increment of treatment temperature and time. It can be attributed to the degradation of the hemicellulose in bamboo cell walls. As we know, hemicellulose contains a large number of free hydroxyl groups. In addition, the number of free hydroxyl groups is greatly decreased due to the increment of treatment temperature and time. Thus, the hygroscopicity decreased. At the same time, the equilibrium moisture content of bamboo was further decreased after the flattening process. During the flattening process, the internal structure of the moso bamboo changes due to the extrusion of the flattening rollers. As analyzed in SEM observation, the parenchyma cells compacted so that the water molecules can hardly enter. Thus, the equilibrium moisture content further decreased compared with that of the softened bamboo samples [40,41,42]. The radial and tangential shrinkage of bamboo samples showed a decreasing tendency. This is also due to the hygroscopicity decreased.

### 3.4. Macro/Micro-Mechanical Properties of Untreated, Softened Treated, and Flattened Bamboo Samples

**Figure 7:**
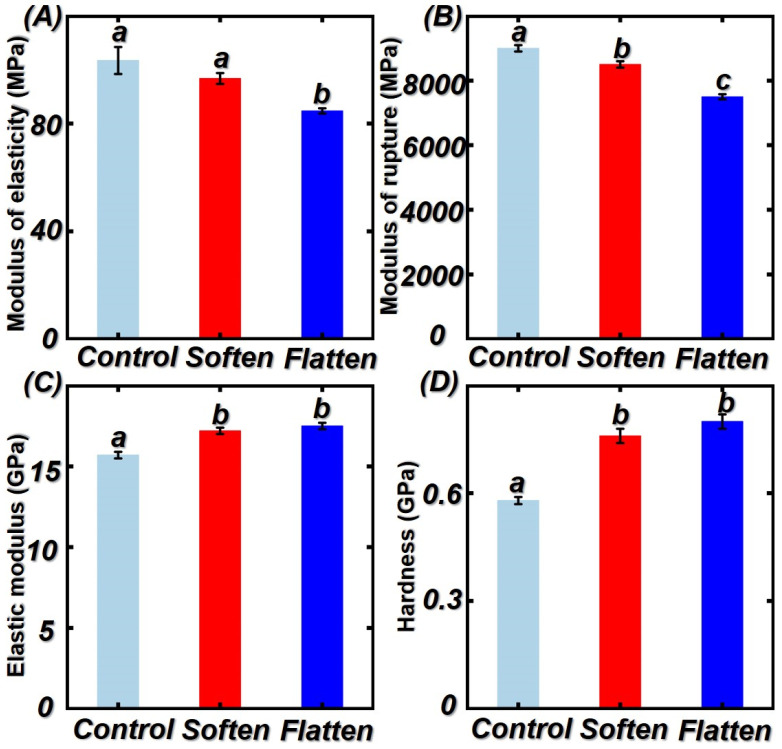
Mechanical properties of control, softened treated, and flattened bamboo samples at 180 degrees and 8 min: (**A**) modulus of elasticity; (**B**) modulus of rupture; (**C**) elastic modulus of bamboo cell walls; (**D**) hardness of bamboo cell walls. Different capital letters represents the significant difference between heat treatment groups (*p* < 0.05). The error bar in the picture represents the standard deviation.

The macro/micro mechanical properties of untreated, softened treated, and flattened bamboo samples are shown in Figure 7. The initial modulus of rupture (MOR) of untreated, softened treated, and flattened bamboo samples were 9000, 8470, and 7500 MPa, respectively. After softening by saturated steam, the MOR decreased by 5.8%, while, when the softened treateded bamboo samples were flattened, the MOR reduced by 16.7%. According to previous literature [40,41,42], firstly, bamboo is softened under the high-pressure and high-temperature condition and then pyrolyzed during the softening process. When the temperature reached 180 degrees Celsius, the hemicellulose decreased significantly due to the saturated steam heat treatment. High treatment temperature had significant effect on the modulus of rupture bamboo. Furthermore, during the flattening process, the flattened rollers make grooves or cuts on the inner surface of the bamboo culms. As SEM analysis, the bamboo fibers were broken and exposed under the action of flattened rollers so that the MOR of flattened bamboo board decreased further in comparison to the softened treated bamboo. Nanoindentation (NI) is a useful approach to access the micro-mechanical properties of bamboo cell walls. In order to analyze the effects of the softening–flattening process on bamboo cell walls, NI was applied for this objective. The elastic modulus and hardness of the untreated bamboo were 15.7 and 0.58 GPa, respectively. Results from statistical analysis demonstrate that the MOE and hardness of soften bamboo increased significantly. It can be attributed to the increased crystallinity and lignin content. According to previous studies [43,44,45,46], the macro-mechanical properties of bamboo samples obtained from the macro-scale stress–strain relationship decreased after the softening–flattening process, while the mechanical properties of bamboo cell walls increased. The decrement of bamboo MOR and MOE is due to the decomposition of chemical composition in bamboo cell walls. The micro-mechanical properties of the bamboo cell wall are affected by cellulose crystallinity, lignin content, moisture content, and so on. Thus, the mechanical properties of the bamboo cell wall play a subordinate role to the macro-mechanical properties [47,48].

## 4. Conclusions

Bamboo longitudinal flattening technology, as a newer bamboo processing method, can effectively improve the utilization rate of bamboo materials. This work accesses the effect of the saturated steam softening process and flattening process on bamboo culms through analyzing the change in the micro-morphology, chemical composition, cellulose crystallinity, physical properties, and micro-mechanical properties of bamboo. When the softening parameter is 180 °C for 6–8 min, crack-free flattened bamboo board can be obtained. The results showed that the parenchyma cell deformed after saturated steam heat treatment due to the degradation of chemical composition in bamboo cell walls. After the flattening process, the parenchyma cell became compacted due to the external force. Results from statistical analysis showed that the CrI and lignin content increased significantly while the hemicellulose and cellulose content decreased. The modulus of rupture of flattened bamboo board decreased from 9000 to 7500 MPa due to the grooves made by the flattening roller. The MOE of flattened bamboo board showed the same decreasing tendency. However, the hardness and modulus of elasticity of untreated bamboo sample increased from 0.58 and 15.7 GPa to 0.8 and 17.5 GPa. The micro-mechanical properties of bamboo samples increased due to the increment of lignin content and CrI. This work provides comprehensive process parameters and a softening–flattening mechanism for the manufacture process of crack-free flatten bamboo board, which can attract more attention from bamboo industry entrepreneur and the researchers in bamboo processing.

## Figures and Tables

**Table 1:** The success rate of flattened bamboo board at different temperatures and times.

Temperature	Time	Number of Specimens	Success Rate for Flattening/%
140	4	10	0
140	6	10	0
140	8	10	0
150	4	10	10
150	6	10	0
150	8	10	0
160	4	10	50
160	6	10	60
160	8	10	60
170	4	10	60
170	6	10	70
170	8	10	70
180	4	10	90
180	6	10	100
180	8	10	100
